# The Potential Strategies of ZnIn_2_S_4_-Based Photocatalysts for the Enhanced Hydrogen Evolution Reaction

**DOI:** 10.3389/fchem.2022.959414

**Published:** 2022-07-12

**Authors:** Meng Tang, Weinan Yin, Feiran Zhang, Xia Liu, Longlu Wang

**Affiliations:** ^1^ College of Electronic and Optical Engineering & College of Flexible Electronics (Future Technology), Nanjing University of Posts and Telecommunications, Nanjing, China; ^2^ College of Chemistry and Chemical Engineering, Qingdao University, Qingdao, China

**Keywords:** ZnIn_2_S_4_, morphology and structural engineering, defects engineering, doping engineering, heterojunction engineering

## Abstract

Photocatalysis is a potential strategy to solve energy and environmental problems. The development of new sustainable photocatalysts is a current topic in the field of photocatalysis. ZnIn_2_S_4_, a visible light-responsive photocatalyst, has attracted extensive research interest in recent years. Due to its suitable band gap, strong chemical stability, durability, and easy synthesis, it is expected to become a new hot spot in the field of photocatalysis in the near future. This mini-review presents a comprehensive summary of the modulation strategies to effectively improve the photocatalytic activity of ZnIn_2_S_4_ such as morphology and structural engineering, defects engineering, doping engineering, and heterojunction engineering. This review aims to provide reference to the proof-of-concept design of highly active ZnIn_2_S_4_-based photocatalysts for the enhanced hydrogen evolution reaction.

## Introduction

Since 1972, when Fujishima and Honda demonstrated that hydrogen can be produced from water by the photoelectrochemical reaction using a TiO_2_ photoelectrode, photocatalytic technology has provided a feasible strategy for hydrogen generation. The key to catalytic hydrogen evolution lies in the development and utilization of catalysts (Zou et al., 2019; Wu et al., 2021; Zhitong Wang et al., 2021; Bhavani et al., 2022). For photocatalytic catalysts, hydrogen is produced by reducing hydrogen ions using electrons and protons generated in sunlight. So far, researchers have developed a variety of types of semiconductor photocatalysts, such as oxide-type semiconductor photocatalysts (Zhang et al., 2017; Zhao Zhang et al., 2018; Idris et al., 2020), nitrogen (oxygen) compound-type semiconductor photocatalysts (Shuqu Zhang et al., 2018; Zhu et al., 2022), and sulfide-type semiconductor photocatalysts (Kuang et al., 2016; Ruijie Yang et al., 2021). Metal sulfide has become one of the most important semiconductor materials due to its excellent visible light response, suitable band gap structure, and low cost (Song et al., 2021; Zhitong Wang et al., 2021; Zhou et al., 2022). However, CdS and CdIn_2_S_4_ still have some obstacles, such as rapid recombination of photogenic electrons and holes, low specific surface area, and photocorrosion. Therefore, it is necessary to determine an effective method to improve the activity and stability of sulfide semiconductors.

Among the semiconductor photocatalysts currently studied, ZnIn_2_S_4_ as one of the ternary metal sulfides has attracted extensive attention due to its narrow band gap, good chemical stability, and strong photoelectric conversion ability. Compared with single metal sulfides (CdS and ZnS etc.), ZnIn_2_S_4_ has more excellent photoelectric characteristics, physical and chemical stability, and environmental friendliness and has greater durability in photocatalytic reactions.

**FIGURE 1:**
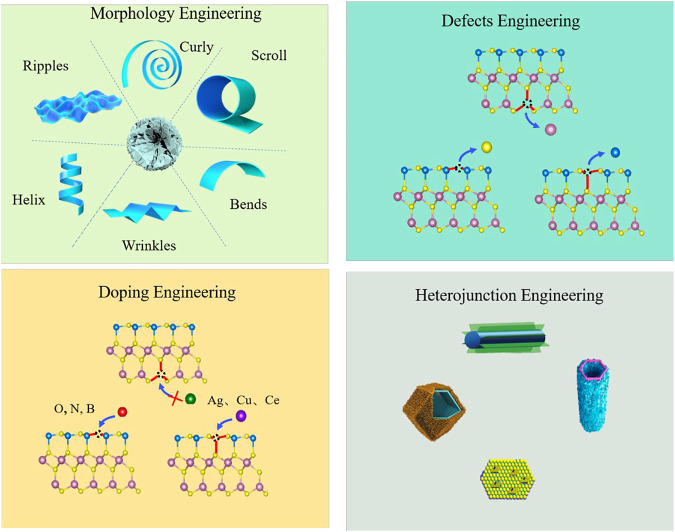
Potential strategies of ZnIn_2_S_4_-based photocatalysts for the enhanced hydrogen evolution reaction.

Compared with other ternary metal sulfides, ZnIn_2_S_4_ is the only AB_2_X_4_ series compound with a layered structure, non-toxic, and has a convenient synthesis process; the unique cone structure has a huge surface area for the photocatalytic reaction and also provides plenty of active sites, which makes ZnIn_2_S_4_ have greater application value in the field of energy conversion (Jie Wang et al., 2021; Man Wang et al., 2021). However, the high photoexcited charge recombination ratio makes ZnIn_2_S_4_ unable to effectively utilize solar energy, and the photocatalytic efficiency is significantly limited (Tu et al., 2018; Chao et al., 2021a; Yijin Wang et al., 2021; Yu et al., 2022). Some potential strategies such as ion doping, morphology regulation, design defects, heterojunction structure design, and loading cocatalyst have been explored (Figure 1).

## Optimization of Photocatalytic Hydrogen Evolution Performance of ZnIn_2_S_4_

### Morphology and Structural Engineering

1. It is well known that precise control of the morphology of semiconductor photocatalysts plays an important role in improving their physical and chemical properties and the performance of photocatalytic systems (Xie et al., 2021; Xuehua Wang et al., 2021; Cheng et al., 2022; Mingming Liu et al., 2022; Wang et al., 2022; Xia Liu et al., 2022). The photocatalytic performance of ZnIn_2_S_4_ photocatalyst can be significantly improved by morphological adjustment, which can be attributed to the following four factors: 1) the specific surface area of ZnIn_2_S_4_ can be increased; 2) it promotes mass transfer and light capture; 3) it is beneficial to expose more active sites on the surface that can participate in redox reactions; 4) it shortens the migration distance and accelerates the migration speed of photogenerated carriers. Therefore, researchers have explored different morphologies of ZnIn_2_S_4_ as a photocatalyst to improve its photocatalytic performance, including nanosheets, nanoflowers, nanowires, nanorods, nanorings, and nanotubes (Guan et al., 2018; Xu et al., 2021).

### Doping Engineering

Element doping can extend the scope of light absorption, add catalytic sites of a photocatalyst, and adjust the hydrogen adsorption and desorption characteristics (Ida et al., 2018; Quan Zhang et al., 2021; Hou et al., 2022). By introducing donor/acceptor energy levels into the doped ions in semiconductors, the concentration and energy distribution of carriers near the conduction band/valence band edge can be adjusted to improve the electron transition behavior. Therefore, with the rapid development of research on photocatalyst modification of ZnIn_2_S_4_, many researchers are committed to introducing cations or anions into ZnIn_2_S_4_.

Cationic doping caused Fermi levels to pass through the conduction band, giving the material metallic properties, thus improving the conductivity and photocarrier migration ability. For example, Qiu et al. (Pan et al., 2021; Qiu et al., 2021; Shi et al., 2022) introduced nickel ions into the ZnIn_2_S_4_ lattice by the solvothermal method and prepared Ni-doped ZnIn_2_S_4_ nanosheets with few layers. The photocatalytic activity of ZnIn_2_S_4_ nanosheets was about seven times higher than that of pure ZnIn_2_S_4_ nanosheets. Theoretical calculations show that Ni ions are preferentially embedded in the zinc rather than indium sites in tetrahedrons, which induced a narrower band gap, higher electronic conductivity, and more charge carriers that can participate in the hydrogen evolution process. More importantly, Ni dopants can subtly change the electronic structure of the S site and achieve the optimal free energy of hydrogen adsorption on Ni by fine-tuning the S−H bond energy. Therefore, the Ni doping in ZnIn_2_S_4_ nanoparticles can prolong the lifetime of the photoexcited charge and then enhance the activity of photocatalytic hydrogen evolution. Therefore, the doping of metal cations in the photocatalyst can improve the light absorption range and contribute to the enhancement of photocatalytic activity. On the contrary, anion (O, N, and P, etc.) doping can regulate the valence band to promote the migration of holes and adjust the conduction band to enhance the reduction ability of photogenerated electrons (Goswami et al., 2021; Shuqu Zhang et al., 2021).

### Defects Engineering

Defects engineering is applied to photocatalysts to improve the separation efficiency of photocarriers. The introduced defects can be used as a center to capture photocarriers and prevent their recombination, thus improving charge separation and exposing more active sites. Vacancies in photocatalysts are typical point defects, which play an important role in improving photocatalytic performance due to their regulation of physicochemical and photoelectrochemical properties such as photocarrier migration, light absorption, surface acidity and alkalinity, surface active sites, adsorption properties, solubility properties, and electronic structure. In recent years, with the increasing interest in ZnIn_2_S_4_ photocatalysts, the studies on vacancy engineering based on ZnIn_2_S_4_ (sulfur, zinc, and indium vacancies) are also increasing (Lee et al., 2019; Pengfei Wang et al., 2019).

Yu Liu et al. (2022) proposed preparing ZnIn_2_S_4_ microspheres with S vacancy defects through solvothermal and low-temperature hydrogenation reduction strategies. Due to the formation of S vacancy defects on the surface, the band gap of ZnIn_2_S_4_ microspheres was reduced to 2.38 eV, which has good visible light response activity. Experimental results and density functional theory calculations showed that the surface S vacancy caused by the surface field potential difference promotes the spatial separation of electrons and holes, thus improving the performance of the photocatalyst and greatly deepening the surface defects engineering understanding of how to affect the separation of light raw charge and to find other efficient and stable metal sulfide photocatalysts which provides a new train of thought. Tai and Zhou, (2021) used reactive ion etching to generate Zn vacancies in ZnIn_2_S_4_ particles. With the increase of Zn vacancy concentration, the band gap of ZnIn_2_S_4_ decreases from 2.17 to 2.06 eV. Under the optimum Zn vacancy concentration, the photocatalytic hydrogen evolution rate of ZnIn_2_S_4_ is 2.7 times higher than that of pure ZnIn_2_S_4_, and the photocatalytic process of ZnIn_2_S_4_ is stable without any degradation through cyclic experiments, showing good stability. The existence of Zn vacancies reduces the charge carrier transfer resistance, improves the charge separation rate, and prolongs the emission decay life (He et al., 2019).

In the sandwich ZnIn_2_S_4_ stacking structure, Zn or S atoms exposed to the surface are easily desorbed, resulting in Zn or S defects. However, due to steric hindrance, the atoms located in the middle layer of a sandwich structure are difficult to be removed, so it is difficult to form in-layer defects. Zn or S defects promote the directional migration of photogenerated electrons but have little effect on hole regulation. Luan et al, (2022) successfully prepared ultra-thin ZnIn_2_S_4_ nanosheets with an abundant [InS]_6_ intermediate layer and perfect [InS]_4_ and [ZnS]_4_ surface layer by controlling the crystal growth of ZnIn_2_S_4_ with the rapid heating and hydrothermal method. The in vacancy induces the redistribution of orbitals near the maximum value of the valence band, separates the oxidation and reduction sites on both sides of the ultra-thin ZnIn_2_S_4_ nanosheet with in vacancy, and increases the density of states between the valence band and the conduction band. The electrons around indium vacancy are delocalized, which is conducive to the interlayer charge transfer and improves the conductivity of the ZnIn_2_S_4_ nanosheet.

### Heterojunction Engineering

In order to overcome the inherent shortcomings of the high electron–hole recombination rate and low utilization rate of a single unmodified semiconductor photocatalyst, the construction of heterojunction by coupling two semiconductor materials is generally considered to be an effective strategy (Yang et al., 2020; Liu et al., 2021; Xu et al., 2022). The construction of heterojunction with a suitable band position will form a potential gradient between the heterogeneous interfaces to promote the separation and transfer of photocarriers and can also enhance the optical capture performance (Zhao et al., 2019; Mu et al., 2020; Zhang et al., 2020; Zuo et al., 2020). According to the band orientation and carrier transfer path, the structure of heterojunction photocatalyst can be divided into many types, including Type−I (transboundary state photocatalyst), Type−II (alternate state photocatalyst), Z-scheme (alternate state photocatalyst), and Mott−Schottky type (Yang et al., 2018; Li et al., 2019; Hu et al., 2020). In recent years, various heterostructures based on ZnIn_2_S_4_ have been successfully constructed, and their photocatalytic properties in energy and environmental applications have been studied (Zhenfei Yang et al., 2021; Chen et al., 2022).

The Type 1 heterojunction is a kind of semiconductor heterojunction in which the valence band and conduction band of one semiconductor are located between the valence band and conduction band of the other one. Under the irradiation of incident light, the conduction band to the electronic from high to low conduction band direction and the hole from low to high with direction, in the process of a photocatalytic oxidation–reduction reaction, will be two semiconductor materials to bring to the lower conduction band and a high price (Chao et al., 2021b; Longlu Wang et al., 2021). Different from Type−I heterojunction, Type−II heterojunction is formed by a staggered conduction band and valence band of two semiconductor materials (Jiang et al., 2019; Yan et al., 2019). The movement direction of charge carriers and redox reaction sites of Type−I heterojunction is the same as that of Type−I heterojunction. Due to structural differences, Type−II heterojunction can effectively promote the separation of photogenerated carriers and inhibit their recombination, and the energy conversion efficiency is significantly improved (Zizhong Zhang et al., 2018; Zhao et al., 2021). Although Type−II heterojunction photocatalysts exhibit good photocatalytic performance, such high photocatalytic performance sacrifices the redox ability of charge carriers, so the reduced driving force may not smoothly drive the specific photocatalytic reaction. Due to the well matching of the electronic band structure of the two semiconductor materials, the Z-scheme heterojunction keeps the electrons at a more negative potential and the holes at a corrected potential, resulting in a strong redox ability (Sabbah et al., 2022; Su et al., 2022).

## Perspectives

This review presents a comprehensive summary of the modulation strategies to effectively improve the photocatalytic activity of ZnIn_2_S_4_ such as morphology and structural engineering, defects engineering, doping engineering, and heterojunction engineering. Although a large number of promising results have been achieved in photocatalytic HER for ZnIn_2_S_4_, there are still many untapped areas to be investigated to realize their full potential. Branched flower-like nanostructures with atomically thin petals are usually obtained by liquid-phase synthesis. This morphology is very favorable for catalysis because it maximizes exposure of active sites, rather than planar stacking like 2D nanosheets. The next key challenge lies in understanding the nucleation stage in the liquid phase so that the platelet topography can be controlled reliably. Further work should be able to develop more precise crystal growth methods and gain full control of defects/doping elements/functional groups to identify, quantify, and ultimately develop active sites. At the same time, complementary advanced characterization techniques, such as nanoscale STM, XAFS, HAADF-STEM, positron annihilation spectroscopy, and ultrafast transient absorption spectroscopy, also need to be developed in parallel to probe the reaction kinetics at the atomic or molecular scale. With the synergistic development of robust and novel ultrathin 2D materials, catalytic hydrogen evolution technology is expected to achieve greater breakthroughs (Hu et al., 2013; Bingqing Wang et al., 2019).
